# The staphylococcal collagen adhesin CNA35 effectively detects collagen and its fragments in blots after SDS-PAGE

**DOI:** 10.1016/j.mbplus.2025.100174

**Published:** 2025-05-15

**Authors:** Elena N. Pokidysheva, Jennifer Diaz Sales, Shinomi Yagi, Tomonori Ueno, Kanako Sasai, Alice Makarenko, Hans Peter Bächinger, Kazunori Mizuno, Sergei P. Boudko

**Affiliations:** a Nephrology Division, Department of Medicine, Vanderbilt University Medical Center, Nashville, TN 37232, USA; b Aspirnaut Program, Vanderbilt University Medical Center, Nashville, TN 37232, USA; c Nippi Research Institute of Biomatrix, Toride, Ibaraki, Japan; d Scool of Science and Engineering, Seattle University, WA, USA; e Research Department, Shriners Hospital for Children, Portland, OR, USA; f Department of Biochemistry and Molecular Biology, Oregon Health & Science University, Portland, OR, USA; g Center for Matrix Biology, Vanderbilt University Medical Center, Nashville, TN 37232, USA; h Biochemistry Department, Vanderbilt University, Nashville, TN 37232, USA

**Keywords:** Collagen, Triple helix, 7S, CNA35 trimer, SDS-PAGE, Western blot

## Abstract

Collagens are a diverse family of proteins present in the extracellular matrix (ECM) of all animals. They play crucial roles in providing structural support to tissues, forming scaffolds for ECM suprastructures, and signaling cells. Certain collagen-binding proteins from pathogenic bacteria, such as CollageN Adhesin (CNA) from *Staphylococcus aureus*, interact with the collagen triple helix to promote host invasion. The extracellular portion of CNA, known as CNA35, which has a molecular weight of 35 kDa, has been used for *in vitro*, *ex vivo*, and *in vivo* staining of various collagen types in tissues.

Detecting various types of collagens necessitates the use of type- and specie-specific antibodies, which typically exhibit weak affinities for the triple helical regions of collagens. Additionally, the fragmentation of collagens can lead to a loss of detection due to the limited number of available epitopes. Furthermore, antibodies can be expensive, require secondary identification methods, and are often suitable for either immunohistochemistry or western blotting. Although successful procedures for staining collagens in tissues have been implemented, the detection of collagens and their fragments using CNA35 has not been reported for protein blots.

In this study, we examined the detection capabilities of a trimeric form of CNA35 for protein blots following SDS-PAGE. We successfully tested collagens I through VI, as well as fragments of collagen IV, under various conditions. Additionally, we investigated the impact of blocking solutions, incubation time, ligand concentration, and CNA35 concentration on sensitivity.

We achieved superior detection of all tested collagens and collagen IV fragments, including the 7S domain, which is a highly crosslinked complex composed of four triple-helical strands. The method we developed serves as a universal tool for detecting collagens and collagen-containing peptides in protein blots. It offers several advantages, including sub-nanogram sensitivity, low cost, and compatibility with standard western blotting techniques.

## Highlights

• Recombinant trimeric form of CNA35 provides more efficient staining of tissues.
• CNA35 probes are suitable for collagen detection in blots after SDS-PAGE.
• CNA35 is compatible with regular western blot staining procedure.
• CNA35 trimer provides sub nanogram sensitivity for collagen bands.
• CNA35 trimer is applicable for collagen detection in crude and protease processed tissue samples.

## Introduction

Collagen stands out as the predominant protein in humans, playing a crucial role in providing structural support to tissues and contributing to the assembly of the extracellular matrix (ECM) [1]. Collagens are essential and widely distributed molecules in tissues of all animals [2]. Collagen is made of three polypeptide chains forming the triple helical structure [3].

*Staphylococcus aureus* is a significant human pathogen that causes infections such as bacterial arthritis [4], osteomyelitis [5], and acute infectious endocarditis [6]. *S. aureus* primarily causes infections in the extracellular space and attaches to various extracellular matrix proteins, *i.e.*, collagen [7], fibronectin [8], fibrinogen [9], laminin [10], bone sialoprotein [11], elastin [12], and vitronectin [13].

The CollageN binding Adhesin (CNA) protein, which is located on the surface of bacteria, consists of several components: an A-region, a B-region that varies in length, a C-terminal portion that includes a site for anchoring to the cell wall, a hydrophobic transmembrane segment, and a short cytoplasmic segment [14]. The collagen binding activity is found in the A-region, which consists of three subdomains: N1, corresponding to residues 31–140; N2, corresponding to residues 141–344; and N3, corresponding to residues 345–531. [15]. The N2 subdomain, which is approximately 19 kDa in size, includes the essential site for collagen binding [16]. In contrast, this central segment has a tenfold lower affinity for collagen than the full-length A-region, indicating that the surrounding sequences play a significant role in collagen binding [14,17]. Interestingly, the protein construct related to the predicted N1 and N2 subdomains (residues 31–344, known as CNA35 [18]) displayed a binding affinity for collagen greater than that of the complete A-region [15].

**Fig. 1:**
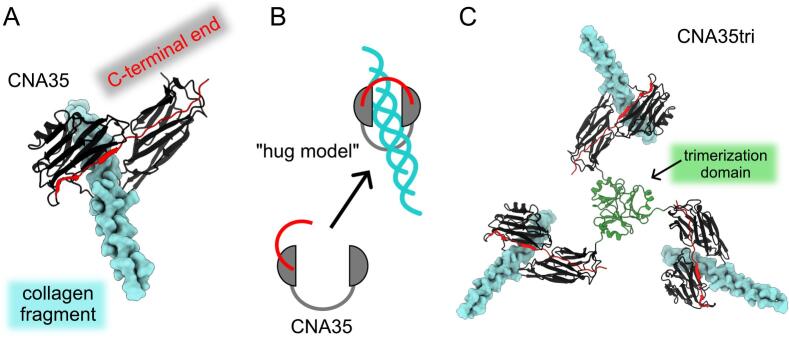
**CNA constructs.** (A) The CNA35 monomer binds to the collagen triple helix. Notably, the C-terminal end (in red) forms a lock that connects one IgG domain to another. This configuration has the potential to interact with and trap triple helical molecules. (B) A “hug model” for collagen binding. (C) CNA35tri was designed as a fusion with the trimerization domain of collagen XVIII, ensuring that the C-terminal end of CNA35 remains in its free configuration. The figures were created using the ChimeraX program (https://www.cgl.ucsf.edu/chimerax/) with PDB models 2f6a and 3hsh. (For interpretation of the references to colour in this figure legend, the reader is referred to the web version of this article.)

The crystal structures of CNA35 in its apo-state and in complex with a collagen peptide (Fig. 1A) have led to the proposal of a dynamic, multistep binding model known as the “Collagen Hug” [15] (Fig. 1B). In this complex, the collagen peptide passes through a circular opening created by the two subdomains and the N1–N2 linker.

CNA35 became a popular probe for visualizing collagen in tissue, live cell culture, and whole organisms [[19], [20], [21], [22]]. Over time the use of CNA35 has been extended to visualizing collagen in fibrotic and scarring tissues, tumor vasculature, and abdominal aorta aneurysm by using such techniques as computer tomography [[23], [24], [25], [26], [27]], magnetic resonance imaging [28], photoacoustic [27], and ultrasound [27,29].

Although the CNA35 probe demonstrates high specificity for collagen, it has a moderate affinity of approximately 0.2 µM [15]. Several studies have successfully developed multimeric forms of CNA35 that show a significantly higher affinity (avidity) by utilizing liposomes [19], micelles [30], and dendrimers [[31], [32], [33]].

As a biochemistry laboratory, we analyze the quality of collagens derived from various sources, including tissue, cell culture, or recombinant production, using SDS-PAGE. When examining crude samples, we are limited to using antibodies for western blots or collagen hybridizing peptides for in-gel western blots [34]. The reagents used are often expensive and can exhibit non-specific binding as well as low sensitivity. When researching collagens in lower organisms, such as *Drosophila* and *C. elegans*, the availability of species-reactive antibodies is often a challenge. Additionally, fragmentation of collagen may result in the loss of binding epitopes. Furthermore, extensive crosslinking of collagen fragments can make them inaccessible to hybridizing peptides. Our goal was to identify a more universal and cost-effective probe for detecting collagen bands in protein gels or blots. In our findings, we discovered that a modified version of CNA35 is effective in detecting collagen bands on blots following the standard SDS-PAGE procedure.

For routine in-house staining of collagen in tissues, we recently designed a trimeric form of CNA35 by fusing it with the collagen XVIII trimerization domain (CNA35tri) (Fig. 1C). This construct was successfully used for staining shark tissues [35]. We found that CNA35tri is a highly effective probe for detecting collagen in protein bands on blots. We developed the protocol to detect sub-nanogram quantities of collagen I.

To demonstrate its capabilities, we applied our new probe to detect collagens I through VI, analyze both crude and pepsin-treated tissue preparations, and identify cleavage fragments of collagen IV. Additionally, the probe is compatible with standard antibodies. Overall, the new variant of CNA35tri serves as a universal collagen probe for western blots.

## Results

### Design of trimeric CNA35

A moderate affinity of approximately 0.2 µM has been reported for CNA35 [15], which could limit the detection capabilities in various collagen detection techniques. Additionally, the monomeric form of CNA35 shows a fast dissociation rate, with a half-life of just a few minutes [31], potentially resulting in a loss of signal under certain conditions. In contrast, the trimeric/tetrameric forms of CNA35 exhibit much higher affinities and remain mostly associated with collagen, even after 8 h of continuous flow washing [31].

To avoid the complex native chemical ligation method used to generate trimeric/tetrameric CNA35, as described in previous studies [31], we chose to design a recombinant version of a CNA35 trimer. This was accomplished using the small (approximately 6 kDa) and efficient trimerization domain of human collagen XVIII [36]. Based on the crystal structure of a CNA35-collagen fragment complex and a proposed “hug model” for binding to the collagen triple helix [15], we created a fusion of the N-terminus of CNA35 with the C-terminus of the trimerization domain. This design keeps the C-terminal end of CNA35 in a free state, facilitating easier attachment and detachment from collagen triple helices. This approach is similar to that of CNA35 probes fused with various fluorescent proteins, which have demonstrated functionality [37].

Both CNA35 and the trimeric CNA35 (CNA35tri) (Fig. S1) were recombinantly produced in E. coli, purified (Fig. S2), and labeled with fluorescent dyes as detailed in the Experimental Procedures.

**Fig. 2:**
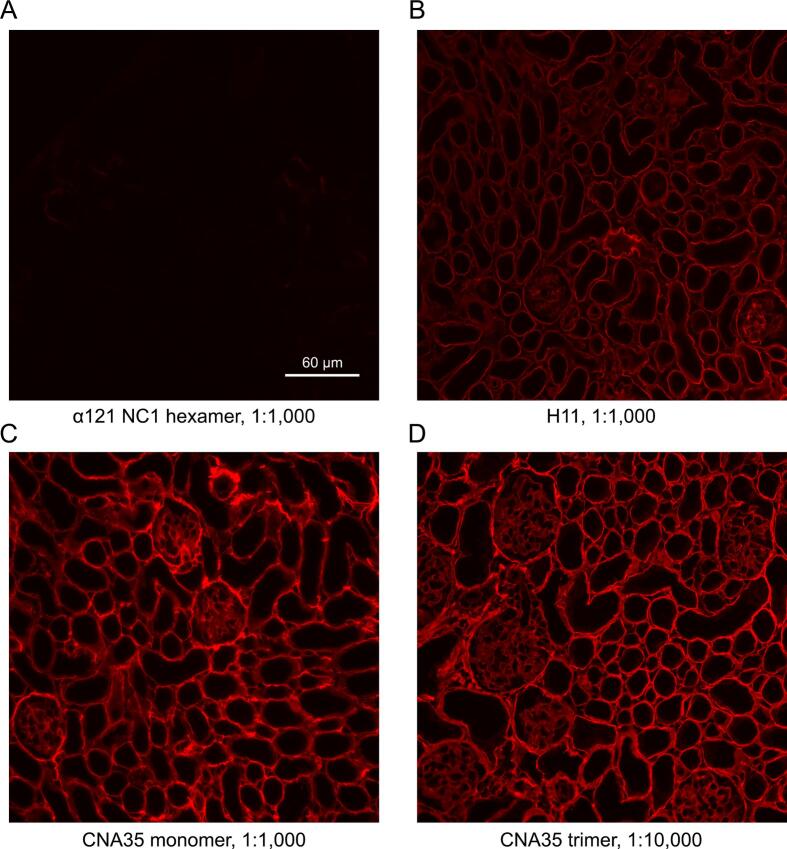
**Staining of kidney tissue with CNA constructs labeled with AF-568.** Negative control: Staining with the NC1 hexamer of collagen IV^α121^, labeled with AF-568. (B) Positive control: Staining with the anti-human alpha 1 (IV) NC1 antibody, clone *H*11 (Chondrex), at a dilution of 1:1,000, followed by staining with a secondary anti-rat antibody conjugated to Alexa Fluor^TM^ 568 (Thermofisher). (C) CNA35-AF568 at 1:1,000 dilution. (D) CNA35tri-AF568 at 1:10,000 dilution. Stock solutions of the probes were at 1 mg/ml.

To demonstrate the functionality of CNA35tri, we performed comparative staining of kidney tissue sections using fluorescently labeled CNA35 and CNA35tri (Fig. 2). We used ten times less CNA35tri to achieve a similar staining intensity.

### Detection of collagen in blots after SDS-PAGE

**Fig. 3:**
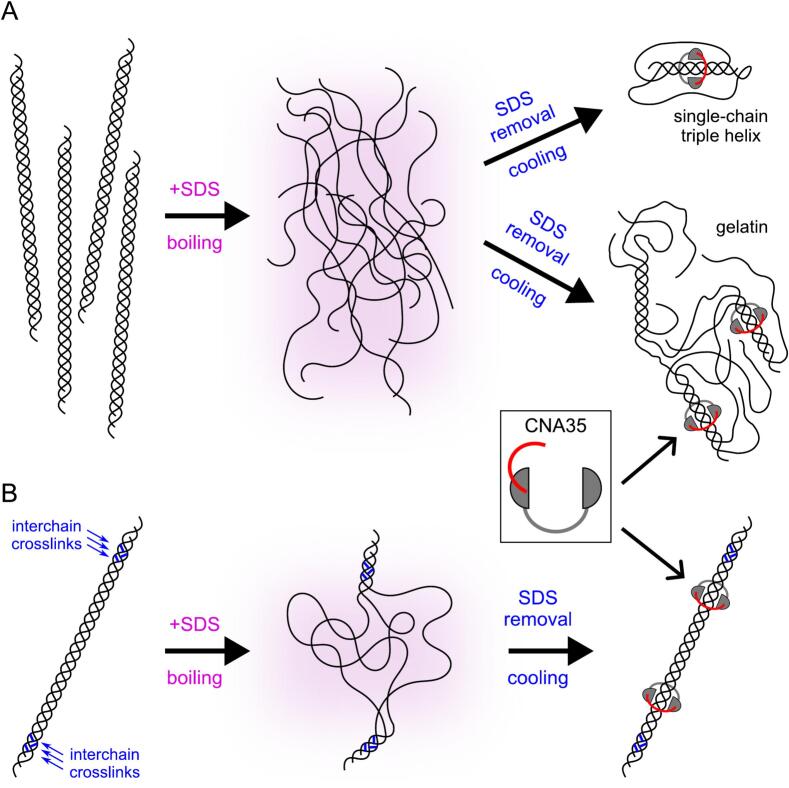
**CNA35 binding to refolded collagen.** (A) Non-crosslinked chains of collagen dissociate into single random coil chains when treated with SDS and heated. Once the SDS is removed, the chains can anneal to form gelatin-like structures with localized segments of the triple helix. (B) Interchain crosslinked collagen molecules are able to restore most of their triple helical structure under favorable conditions. In both scenarios, CNA35 molecules are expected to bind to either partially or fully renatured triple helical structures.

The collagen triple helix serves as a substrate for CNA35 binding. However, treatment with SDS and boiling the samples before running them on SDS-PAGE denatures the collagen. After transferring the denatured collagen molecules onto the blot and washing away SDS, certain renaturation steps may enable the reformation of local segments of the triple helix (Fig. 3A). This reformation can be enhanced when collagen chains are crosslinked (Fig. 3B). The renatured fragments of the triple helix can then serve as substrates for CNA35.

**Fig. 4:**
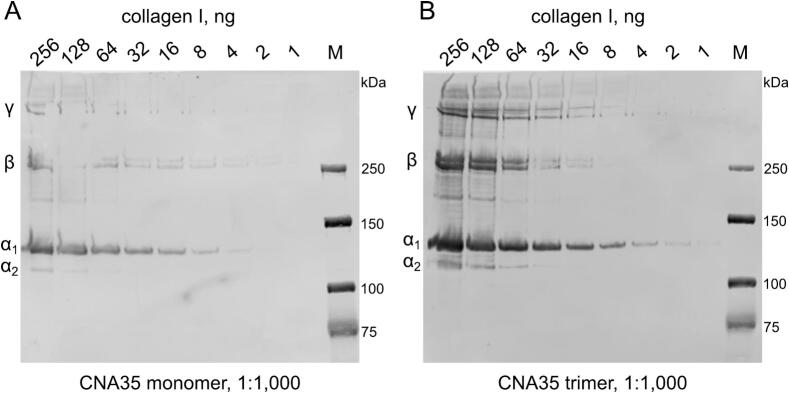
**Detection of collagen I with CNA35 and CNA35tri probes after SDS-PAGE.** (A) The CNA35-IR800 probe. (B) The CNA35tri-IR800 probe. 6 % SDS-PAGE gels were transferred onto 0.45 µm nitrocellulose membranes and thoroughly washed with water to eliminate SDS. A 5 % non-fat milk solution was used as the blocking agent. For staining, a solution of 1 % non-fat milk in TBS-T was prepared. Probes were added at a concentration of 0.75 μg/ml and incubated at room temperature for 1 h.

We conducted a serial dilution of collagen I and utilized fluorescently labeled CNA35 and CNA35tri to determine whether the collagen chains could be detected on a blot after SDS-PAGE following the standard protocol described in the Experimental Procedures. To ensure proper binding of CNA35 to the collagen triple helix, it is important to allow sufficient time for the refolding of denatured collagen chains after blotting and removing SDS. Our results showed that the α, β, and γ chains of collagen I were detectable with these probes (Fig. 4). Notably, the CNA35tri probe demonstrated stronger detection of collagen chains and greater sensitivity for diluted samples.

**Fig. 5:**
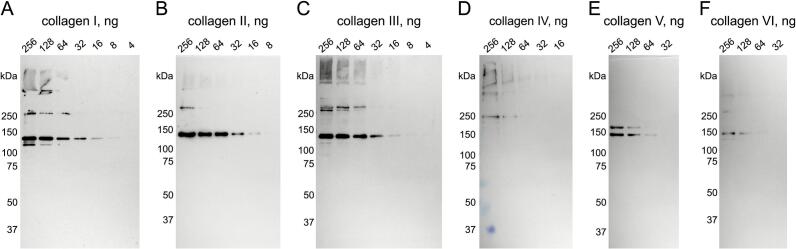
**Detection of collagen types I through VI using CNA35tri-IR800 probe.** (A) Collagen I. Pepsin solubilized from bovine skin. (B) Collagen II. Pepsin solubilized from porcine cartilage. (C) Collagen III. Pepsin solubilized from bovine skin. (D) Collagen IV. Acid solubilized from porcine lens capsule. (E) Collagen V. Pepsin solubilized from porcine placenta. (F) Collagen VI. Intact extraction from porcine cornea. 7.5% SDS-PAGE gels were used to separate protein bands under reducing conditions. The standard staining protocol was used. Data was collected using the Amersham™ ImageQuant 800 CCD imager (Cytiva).

We also confirmed that CNA35tri is an effective probe for detecting various types of collagens, specifically types II through VI (Fig. 5). Consequently, CNA35 serves as a universal probe for full-length collagens in blots following SDS-PAGE.

### Development of advanced protocol

We conducted a series of experiments aimed at improving the sensitivity of CNA35tri for staining collagen chains in blots. Our focus was on varying several factors, including the type of blocking solution, staining duration, incubation temperature, and probe dilution.

Our results indicated that extending the staining time to overnight at 4 °C, compared to 1 h at room temperature, significantly enhanced the signal and detection limit. Additionally, we discovered that non-fat soy protein was more effective than non-fat milk and other animal-based blocking solutions.

**Fig. 6:**
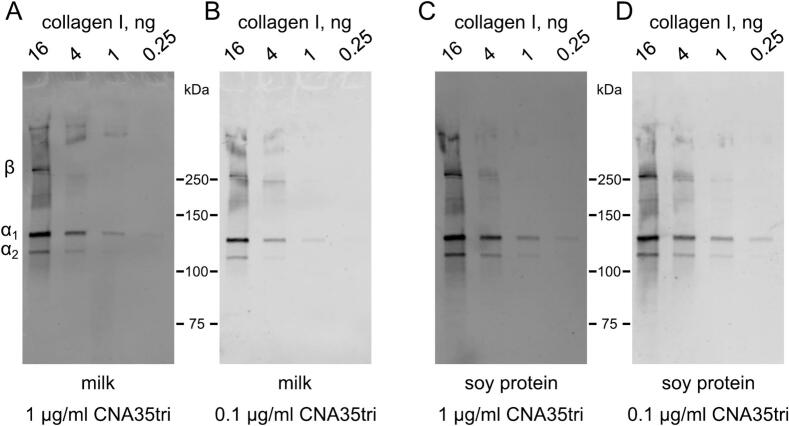
**Protocol improvement.** The Effect of Blocking Agents and Probe Concentration on Sensitivity and Background. 7.5 % SDS-PAGE gels were used to separate protein bands under non-reducing conditions. Membranes were blocked using either 5 % non-fat milk (A and B) or hot 5 % soy protein with 1 % SDS (C and D). The CNA35tri-IRDye800 probe was applied at concentrations of 1 μg/ml (A and C) or 0.1 μg/ml (B and D). Staining solutions consisted of either 1 % non-fat milk (A and B) or 1 % soy protein (C and D) in TBS-T buffer. Blots were scanned at the same time, and brightness and contrast were adjusted equally to emphasize differences in the background. Results indicated that using soy protein with the lowest probe concentration provided the best sensitivity and lowest background.

Ultimately, we developed an advanced staining protocol, described in the Experimental Procedures, which utilizes a soy protein blocking solution along with a highly diluted CNA35tri (see Fig. 6).

### Analysis of collagen in tissue homogenates

**Fig. 7:**
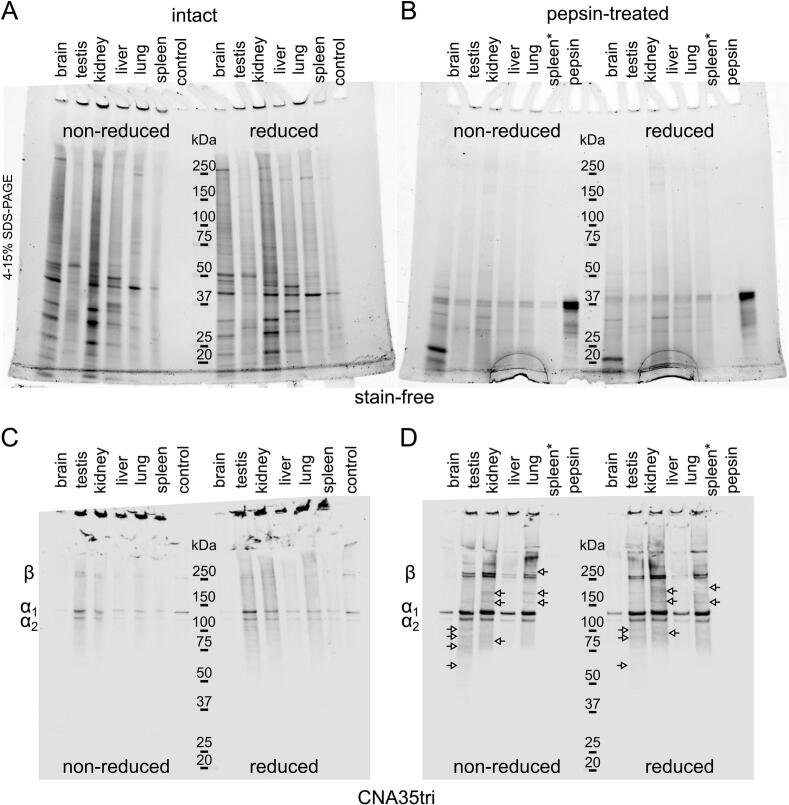
**Analysis of collagen content in intact and pepsin-treated whole organ homogenates.** Murine organs were homogenized and analyzed in two forms: as intact insoluble homogenates (A and C) and after treatment with pepsin without separating soluble and insoluble fractions (B and D). The amount loaded per lane corresponds to 0.1 mg of the pelleted material. Control – 3.6 ng of collagen I per lane. Amount of pepsin in samples was 0.1 μg. Pepsin only as a control – 1 μg per lane. Whereas collagen I is the major type detected in the blot there are other minor bands (indicated with arrows) that can belong to other types of collagens or their fragments. 4–15 % SDS-PAGE gels were utilized to separate protein bands, and an advanced staining protocol was employed. Spleen* − material clumped after adding acetic acid and could not be suspended for analysis, only soluble fraction was analyzed.

**Fig. 8:**
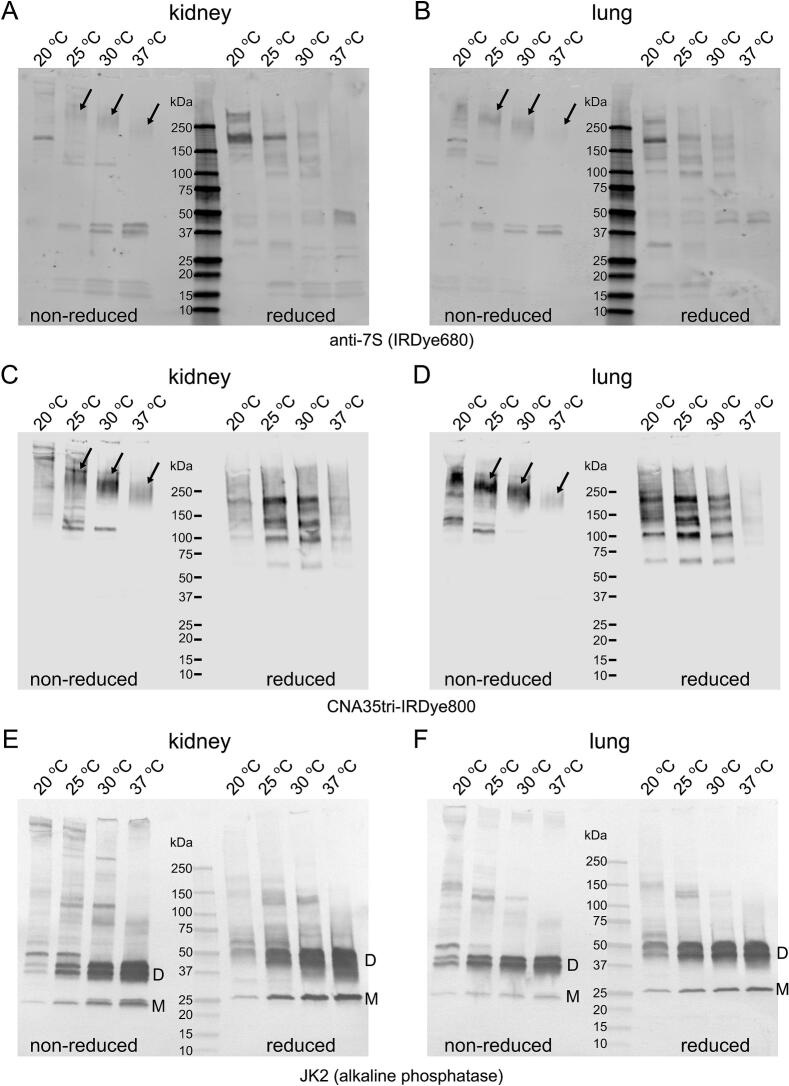
**Collagenase digest of kidney and lung matrix.** Insoluble homogenate of murine kidney and lung was subjected to collagenase digest in a suspension at various temperatures and soluble fractions were analyzed on western blots to detect 7S domain (A and B), collagen triple helix (C and D), and the NC1 domain (E and F). A, C, and E show results for kidney material. B, D, and F show results for lung material. Amount of collagenase added was 12 units/ml. Amount of material loaded per lane corresponded to 2 mg of initial insoluble homogenate. Arrows indicate 7S domain. Under reducing conditions, the 7S domain breaks down into a series of polypeptides that are crosslinked by non-reducible lysyl-derived bonds. NC1 monomer (designated “M”) and dimer (designated “D”) bands are labeled. Anti-7S antibody was C0157 (AssayBioTech). Anti-NC1 antibody was JK2 (from Dr. Y. Sado). 4–15 % SDS-PAGE gels were utilized to separate protein bands, and an advanced staining protocol was employed.

We conducted an analysis of collagen content in various organs collected from a wild-type 3-month old male mouse. The homogenates were cleared of any soluble material and analyzed using blots, either directly or after treatment with pepsin (Fig. 7). Details regarding the amounts of starting and final materials loaded onto the gels are provided in Table 1. The samples showed staining of α, β, and γ chains of collagen I, as well as higher-order crosslinked materials. In contrast, the pepsin-treated samples displayed more abundant bands with lower molecular weight (α, β, γ) and significantly weaker staining of collagen at the gel entry border. This finding highlights the release of collagen molecules from heavily crosslinked collagen fibrils due to the cleavage of crosslinked telopeptides (Fig. 8).

**Table 1:** **Quantity of material used for collagen analysis in murine organs**. Murine organs or their fragments were excised from a mouse and processed as outlined in the Experimental Procedures. The corresponding amounts of insoluble matrix and raw tissues loaded onto the SDS gel are provided for reference.

	Weight of raw tissue (mg)	Weight of insoluble wet pellet after homogenization (mg)	Ratio of pellet to raw material (%)	Amount of insoluble matrix loaded onto a gel (both untreated and pepsin-treated) (mg)	Initial weight of raw tissue processed and loaded onto a gel for untreated and pepsin-treated samples (mg)	The initial amount of insoluble matrix processed with collagenase and loaded onto a gel (mg)	The initial amount of raw tissue processed with collagenase and added to the gel (mg)
brain	395	85	21	0.1	0.5	2.0	9.3
kidney	239	39	16	0.1	0.6	2.0	12.3
liver	250	11	4	0.1	2.4	2.0	47.2
lung	235	80	34	0.1	0.3	2.0	5.9
spleen	123	25	20	0.1	0.5	2.0	9.8
testis	240	21	9	0.1	1.1	2.0	22.5

We used tissue homogenates and treated them with bacterial collagenase at various temperatures. Collagenase is commonly employed to break down the collagen triple helix, which helps release non-collagenous domains as well as heavily crosslinked fragments of collagen. In basement membrane (BM)-rich organs, collagenase treatment releases fragments of collagen IV, the primary component of the basement membrane. Two significant fragments released from the collagen IV scaffold are NC1 and 7S [38].

NC1 is a globular non-collagenous domain, while 7S is a complex formed by the N-terminal ends of four trimeric collagen IV molecules, stabilized by several disulfide and lysyl-derived interchain crosslinks [[39], [40], [41], [42]]. Notably, 7S includes a triple helical fragment that is resistant to collagenase digestion due to the presence of these crosslinks.

We analyzed collagenase digests from the kidney, lung, brain, liver, testis, and spleen (see Figs. 8, S3, S4). Remarkably, CNA35tri outperformed the anti-7S antibody in detecting 7S in both non-reduced and reduced forms. We included data on collagenase digests from the testis and spleen (Fig. S4) to highlight that crude extractions can pose challenges for certain organs. The materials from these tissues contain larger molecular substances that interfere with gel resolution and make it difficult to detect collagenous bands. Therefore, additional purification steps may be necessary for some organs to address this issue.

## Discussion

We believe this is the first reported example of utilizing a collagen-binding protein to detect collagen chains in a blot after performing SDS-PAGE. Rather than relying on expensive and often non-specific antibodies for collagen detection, we present an effective and affordable alternative that utilizes a recombinant protein. This probe can be produced and labeled in laboratories experienced in the recombinant expression of proteins in bacteria. Furthermore, the standard and advanced protocols can be adapted to use regular antibodies for western blotting.

This probe will be particularly beneficial for researchers studying collagen types and fragments for which no antibodies are available, including studies involving lower organisms, for which mammalian-specific antibodies are typically ineffective.

Our approach complements the use of collagen hybridizing peptide (CHP), which is employed to detect unfolded collagen chains in gel [34]. We believe that our method offers a superior opportunity to detect heavily cross-linked chains, which can be challenging to expose for CHP. Furthermore, our method has shown sub-nanogram sensitivity in detecting full-length, non-crosslinked collagen I chains, while CHP detection requires over 10 ng.

Both CNA35 and CNA35tri are capable of detecting collagen chains in the blot; however, the trimeric version exhibits superior performance compared to the monomeric form. Notably, the recommended amount of CNA35tri for reliable detection is ten times less than that of CNA35. The CNA35tri maintains its trimeric structure even at very low concentrations, thanks to the incorporation of the collagen XVIII trimerization domain, which has a midpoint dissociation concentration of approximately 60 pM per chain [36]. At a concentration of 0.1 μg/ml, the molar concentration of CNA35tri is around 800 pM.

CNA35 offers a versatile approach to staining collagen in blots following SDS-PAGE, enhancing the range of detection compounds available for western blotting. Recombinant CNA35 serves as a cost-effective alternative to traditional antibodies and CHP, while also providing unique properties that complement the benefits of other reagents. Collagen molecules still possess many mysteries [3], which can hopefully be unraveled at least in part by using CNA35tri.

## Experimental procedures

### Production of CNA35 constructs

CNA35 synthetic DNA, with optimized codons for *E. coli* (Integrated DNA Technologies), was cloned into the pET23-His-Trx-thr vector, either with or without the sequence for the human collagen XVIII trimerization domain [36]. The cloning was performed using the BamHI and EcoRI restriction sites [43]. The resulting constructs encoded a thrombin-cleavable (thr) His-tagged thioredoxin domain (Trx), which was added either directly to the CNA35 sequence or via the collagen XVIII trimerization domain (18TD). The protein sequences of the constructs are depicted in Fig. S1. The plasmid sequences were confirmed through Sanger sequencing.

The proteins His_6_-Trx-thr-CNA35 and His_6_-Trx-thr-18TD-CNA35 were expressed in the BL21(DE3) strain of E. coli using 1 mM IPTG for induction for 3 h at 37 °C. Cells from 0.5 L of culture were harvested by centrifugation, resuspended in 50 ml of 20 mM Tris-HCl (pH 7.5), and disrupted by ultrasonication on ice. A 30 % stock solution of streptomycin sulfate was added to achieve a final concentration of 1 %, while stirring on ice. The mixture was incubated for 15 min to precipitate genomic DNA. Insoluble material was removed by centrifugation at 10,000 g at 4 °C for 30 min, and the supernatants were adjusted to contain 50 mM sodium phosphate (pH 8), 300 mM NaCl, and 10 mM imidazole.

The His-tagged chimera proteins were affinity purified using columns packed with 5 ml of Ni-NTA resin (Qiagen). The columns were equilibrated with five volumes of buffer containing 50 mM sodium phosphate (pH 8), 300 mM NaCl, and 10 mM imidazole. The supernatants were loaded onto the individual columns, washed with 20 ml of the same buffer containing 20 mM imidazole, and then eluted with 20 ml of buffer containing 500 mM imidazole.

The proteins were dialyzed against Tris-buffered saline (TBS) and subjected to proteolytic cleavage of the thioredoxin domain (Fig. S2A). The monomeric construct was efficiently cleaved with 1 U/ml of thrombin overnight at room temperature, while the trimeric form was resistant to thrombin cleavage, as previously reported [35]. Instead of following the month-long thrombin cleavage method, we found that the trimeric protein could be effectively cleaved by 1 µg/ml of trypsin at 4 °C within four days. The His-tagged thioredoxin was then separated using the same Ni-NTA column, but in this instance, the monomeric and trimeric CNA35 were collected in the flow-through.

The proteins were further purified using anion-exchange chromatography with a Q-sepharose HP column (Cytiva) operated at 20 mM Tris-HCl (pH 8) with a linear gradient of NaCl. The proteins eluted in the range of 10–20 mM NaCl. The fractions were pooled and finally subjected to size-exclusion chromatography (Fig. S2B) using the Superdex Increase column (Cytiva), which was equilibrated with coupling buffer (100 mM sodium carbonate, pH 8.8).

The NC1 hexamer of collagen IV^α121^ was prepared as previously described [44] and used as a negative control for tissue staining.

AF-568 NHS ester fluorophore (Lumiprobe) was used for coupling at a protein concentration of 2 mg/ml at 4 °C overnight. The coupling was performed using a dye-to-protein ratio of 1 mg of dye per 10 mg of protein in a 0.1 M sodium bicarbonate buffer at pH 8.5. Following the reaction, the labeled proteins were separated from any unreacted dye by desalting into PBS buffer. The stock solutions were then adjusted to a final concentration of 1 mg/ml. The resulting constructs, CNA35-AF568, CNA35tri-AF568, and α121NC1hex-AF568 were used for staining frozen tissue sections, and images were captured using fluorescent microscopy.

IRDye® 800CW NHS ester infrared dye (LI-COR) was utilized for coupling at a protein concentration of 1 mg/ml for 2 h at 20 °C. This reaction employed a dye-to-protein ratio of 0.5 mg of dye per 12 mg of protein in a 0.1 M sodium bicarbonate buffer at pH 8.5. Afterward, the labeled proteins were separated from unreacted dye by desalting into PBS buffer. The stock solutions were then adjusted to a final concentration of 0.75 mg/ml. The resulting constructs, CNA35-IR800 and CNA35tri-IR800, were used for collagen band staining in blots, which were subsequently detected using the Odyssey CLx Infrared Imager (LI-COR).

### Fluorescent staining of frozen kidney sections

Mouse kidneys were snap-frozen in OCT immediately after isolation. A cryostat (Leica CM 1950) was used to cut 7 μm sections. The sections were then mounted on glass slides and allowed to air dry for 15 to 30 min at room temperature. After drying, they were fixed in acetone at − 20 °C for 10 min and washed three times with a buffer of 50 mM Tris (pH 7.5), 150 mM NaCl, and 0.05 % Tween-20 (TBS-T). Blocking was conducted with 10 % goat serum (Invitrogen, 50062Z) for 1 h. The sections were incubated with either CNA35-AF568 or CNA35tri-AF568 at dilutions of 1:1,000 and 1:10,000, respectively (these dilutions were prepared from a 1 mg/ml stock solutions) in 1 % goat serum TBS-T at room temperature for 1 h. Following this, the sections were washed three times for 15 min each with TBS-T and then mounted with an antifade mounting solution containing DAPI. Images were captured using an Eclipse Ti microscope (Nikon).

### Sources of various collagen types

Collagen I from rat tail tendon (Corning, 354236) with a reported concentration of 3.61 mg/ml was used for developing the staining protocols described below. The following collection of purified collagens (Nippi Research Institute of Biomatrix) was used to confirm the universal binding of CNA35tri in blots: collagen I, pepsin solubilized from bovine skin; collagen II, pepsin solubilized from porcine cartilage; collagen III, pepsin solubilized from bovine skin; collagen IV, acid solubilized from porcine lens capsule; collagen V, pepsin solubilized from porcine placenta; collagen VI, intact extraction from porcine cornea. The stock concentrations of collagens were determined by the micro-Biuret analysis [45]. Calibration curves were prepared at 0.1 mg/ml − 1 mg/ml using a standard collagen solution. The concentration of the standard collagen solution was quantified by amino acid analysis.

### Running SDS-PAGE gels and transfer.

We used either self-made 6 % SDS mini-gels or 4–15 % Mini-PROTEAN® TGX Stain-Free™ Protein Gels (Bio-Rad). Stain-Free™ Gels were visualized using ChemiDoc XRS + System (Bio-Rad) before transfer. The transfer onto 0.45 µm nitrocellulose membrane (Bio-Rad) was conducted using the Trans-Blot Turbo Transfer System (Bio-Rad). We used programmed pre-settings of the Turbo protocol: 1.3 A, 25 V, for 7 min for one mini gel, and 2.5 A, 25 V, for 7 min for two mini gels.

### Standard protocol for blot staining

#### Reagents and materials

Blocking solution: 5 % w/v non-fat dry milk dissolved in water.

Washing solution: 50 mM Tris (pH 7.5), 150 mM NaCl, and 0.05 % Tween-20 (TBS-T).

Staining solution: dilutions of CNA35tri-IR800 (0.75 mg/ml stock) at a ratio of 1:1,000 to 1:10,000 in 1 % w/v non-fat dry milk dissolved in TBS-T.

### Procedure

To ensure proper binding of CNA35 to the collagen triple helix, it is important to allow sufficient time for the refolding of denatured collagen chains after blotting and removing SDS. This is achieved by incubating the blot for one hour at room temperature or overnight at 4 °C.

1. Transfer the gel, after SDS-PAGE, onto a nitrocellulose membrane (either 0.22 or 0.45 µm pore size is acceptable; however, we do not recommend using PVDF membranes due to poor signal and background staining).
2. Rinse the membrane three times with water for 5 min each time while shaking.
3. Block the membrane for 1 h using the Blocking solution. It also enables the refolding of collagen chains.
4. Incubate the membrane in the Staining solution for 1 h at room temperature or overnight at 4 °C. Prolonged incubation at lower temperatures may enhance the signal. This step also allows additional time for the collagen to refold.
5. Rinse the membrane three times with TBS-T for 5 min each time.
6. Scan the membrane using an appropriate blot imager for detection.

### Advanced protocol for blot staining

#### Reagents and materials

Blocking solution: thoroughly resuspend 5 % w/v Soy Protein Isolate (MP Biomedicals, Catalog Number: 905456) in water. We found that ultrasonication significantly improves the resuspension process. Add 1 % SDS to the solution and boil it to enhance the enrichment of soluble and denatured proteins. After boiling, chill the solution to room temperature, then centrifuge at 10,000 g for 10 min to remove insoluble particles. This step is essential to prevent the adsorption of insoluble particulates to the membrane, which can cause background staining.

Washing solution: 50 mM Tris (pH 7.5), 150 mM NaCl, and 0.05 % Tween-20 (TBS-T).

Staining buffer: mix 1 part of the 5 % soy protein suspension in water with 4 parts of TBS-T. It is recommended to remove any insoluble particles by centrifugation at 10,000 g for 10 min.

Staining solution: dilute the CNA35tri-IR800 (0.75 mg/ml stock) to a final concentration of 1:10,000 in the staining buffer.

### Procedure

To ensure proper binding of CNA35 to the collagen triple helix, it is important to allow sufficient time for the refolding of denatured collagen chains after blotting and removing SDS. This is achieved by incubating the blot overnight at 4 °C during the staining step.

1. Transfer the gel after SDS-PAGE onto a 0.22 or 0.45 µm nitrocellulose membrane. We did not observe any significant differences between the two pore sizes; however, we do not recommend using PVDF membranes, as we were unable to achieve good signal quality and reasonable background staining.
2. Re-boil the Blocking solution. Apply the hot Blocking solution directly to the membrane and incubate it for 1 h at room temperature.
3. Thoroughly rinse both the membrane and the membrane box with plenty of water to remove any residues and splashes of SDS-containing blocking buffer.
4. Wash the membrane three times with water for 5 min each time to remove any remaining SDS. After that, rinse the membrane with TBS-T for extra 5 min.
5. Incubate the membrane in the Staining solution overnight at 4 °C. It also enables the refolding of collagen chains.
6. Rinse the membrane three times with TBS-T for 5 min each time.
7. Scan the membrane using an appropriate blot imager for detection.

### Tissue analysis

Murine organs were collected according to the animal protocol (M1900063-01) approved by the VUMC IACUC. The organs were homogenized using metal beads in T-PER Tissue Protein Extraction Reagent (Thermo Fisher Scientific), which was supplemented with 25 mM EDTA. Three rounds of homogenization were performed, with 3 volumes of the reagent used for every weight of tissue (3 ml per gram). After each round of homogenization, the samples were centrifuged at 14,000 g for 10 min, and the soluble fractions were discarded. Two additional rounds of homogenization and soluble fraction removal were conducted using TBS to eliminate the detergent and EDTA. The resulting pellets were then resuspended in 1.5 volumes of TBS and transferred to a fresh tube to remove the metal beads. The suspensions were ultrasonicated on ice using a microtip, centrifuged to pellet insoluble material, and the supernatant was discarded. This last procedure was repeated once more. The final pellets were weighed, resuspended in cold water at a concentration of 100 mg/ml, and stored frozen at −80 °C for further analysis.

Suspensions were analyzed both with and without treatment using pepsin. For the untreated samples, 1 μl of the suspension was added to 20 μl of 1x SDS-loading buffer, with or without DTT (reduced and non-reduced conditions). The mixture was boiled and loaded into each lane of the gel. For the pepsin treatment, the sample suspension in water was adjusted by adding 0.1 M acetic acid and 0.1 mg/ml pepsin. It was incubated at 20 °C overnight while being vigorously shaken. A volume corresponding to 1 µl of the initial suspension was then run on SDS-PAGE for analysis. The total suspension after pepsin treatment was used without removing any insoluble material. The gels were subsequently blotted and analyzed following the advanced protocol.

For collagenase treatment, the suspensions were prepared by adding 50 mM HEPES (pH 7.5), 10 mM CaCl_2_, 0.02 % w/v sodium azide, and 12 U/ml of bacterial collagenase (Worthington Biochemical, CLSPA grade, 1000 U/mg). The samples were then incubated overnight at various temperatures while being vigorously shaken. After incubation, the samples were centrifuged at 14,000 g for 10 min to collect the soluble fractions. Finally, 20 μl of each sample was analyzed following the advanced protocol.

## Data and materials availability

The DNA sequences of the constructs are available by request. All other data are contained within the manuscript or supporting information. The plasmids are available for sharing upon reasonable requests.

## Funding and additional information

This work was supported by grants R01DK018381 and R01DK131101 from the National Institutes of Health (NIH) awarded to S.P.B. The content of this article is solely the responsibility of the authors and does not necessarily reflect the official views of the NIH. Additionally, J.D.S. received support from the Aspirnaut™ Program, which is partly funded by NIH grant R25DK096999 awarded to Billy G. Hudson.

Molecular graphics performed with 10.13039/100008069UCSF ChimeraX [[46], [47], [48]], developed by the Resource for Biocomputing, Visualization, and Informatics at the 10.13039/100008069University of California, San Francisco, with support from 10.13039/100000002National Institutes of Health R01-GM129325 and the Office of Cyber Infrastructure and Computational Biology, 10.13039/100000060National Institute of Allergy and Infectious Diseases.

## CRediT authorship contribution statement

**Elena N. Pokidysheva:** Writing – review & editing, Investigation, Data curation. **Jennifer Diaz Sales:** Investigation. **Shinomi Yagi:** Investigation. **Tomonori Ueno:** Investigation. **Kanako Sasai:** Investigation. **Alice Makarenko:** Investigation. **Hans Peter Bächinger:** Writing – review & editing, Resources. **Kazunori Mizuno:** Writing – review & editing, Resources, Data curation. **Sergei P. Boudko:** Writing – review & editing, Writing – original draft, Visualization, Validation, Supervision, Resources, Project administration, Methodology, Investigation, Funding acquisition, Formal analysis, Data curation, Conceptualization.

## Declaration of generative ai and ai-assisted technologies in the writing process

While preparing this work, the authors utilized generative AI tools from Grammarly to enhance grammar and readability. After employing these tools, the authors carefully reviewed and edited the content as necessary, taking full responsibility for the final publication.

## Declaration of competing interest

The authors declare that they have no known competing financial interests or personal relationships that could have appeared to influence the work reported in this paper.

## Data Availability

Data will be made available on request.

## References

[1] Ricard-Blum S. (2011). *The collagen family*. Cold Spring Harbor Perspectives in Biology.

[2] Fidler A.L. (2018). *The triple helix of collagens - an ancient protein structure that enabled animal multicellularity and tissue evolution*. Journal of Cell Science.

[3] Bächinger H.P., Boudko S.P. (2025). *Mysteries of the Collagen Triple Helix*. Matrix Biology.

[4] Goldenberg D.L., Reed J.I. (1985). *Bacterial arthritis*. The New England Journal of Medicine.

[5] Waldvogel F.A., Papageorgiou P.S. (1980). *Osteomyelitis: the past decade*. The New England Journal of Medicine.

[6] Pelletier, L.L., Jr. and R.G. Petersdorf, *Infective endocarditis: a review of 125 cases from the University of Washington Hospitals, 1963-72.* Medicine (Baltimore), 1977. **56**(4): pp. 287-313. 875718

[7] Speziale P. (1986). *Binding of collagen to Staphylococcus aureus Cowan 1*. Journal of Bacteriology.

[8] Kuusela P. (1978). *Fibronectin binds to Staphylococcus aureus*. Nature.

[9] Hawiger J. (1982). *Identification of a region of human fibrinogen interacting with staphylococcal clumping factor*. Biochemistry.

[10] Lopes J.D., dos Reis M., Brentani R.R. (1985). *Presence of laminin receptors in Staphylococcus aureus*. Science.

[11] Ryden C. (1989). *Specific binding of bone sialoprotein to Staphylococcus aureus isolated from patients with osteomyelitis*. European Journal of Biochemistry.

[12] Park P.W. (1991). *Binding of elastin to Staphylococcus aureus*. The Journal of Biological Chemistry.

[13] Chhatwal G.S. (1987). *Specific binding of the human S protein (vitronectin) to streptococci, Staphylococcus aureus, and Escherichia coli*. Infection and Immunity.

[14] Patti J.M. (1992). *Molecular characterization and expression of a gene encoding a Staphylococcus aureus collagen adhesin*. The Journal of Biological Chemistry.

[15] Zong Y. (2005). *A 'Collagen Hug' model for Staphylococcus aureus CNA binding to collagen*. The EMBO Journal.

[16] Symersky J. (1997). *Structure of the collagen-binding domain from a Staphylococcus aureus adhesin*. Nature Structural Biology.

[17] Xu Y. (2004). *Identification and biochemical characterization of two novel collagen binding MSCRAMMs of Bacillus anthracis*. The Journal of Biological Chemistry.

[18] Xu Y. (2004). *Virulence potential of the staphylococcal adhesin CNA in experimental arthritis is determined by its affinity for collagen*. The Journal of Infectious Diseases.

[19] Reulen S.W. (2007). *Protein-liposome conjugates using cysteine-lipids and native chemical ligation*. Bioconjugate Chemistry.

[20] Megens R.T. (2007). *Imaging collagen in intact viable healthy and atherosclerotic arteries using fluorescently labeled CNA35 and two-photon laser scanning microscopy*. Molecular Imaging.

[21] Krahn K.N. (2006). *Fluorescently labeled collagen binding proteins allow specific visualization of collagen in tissues and live cell culture*. Analytical Biochemistry.

[22] Boerboom R.A. (2007). *High resolution imaging of collagen organisation and synthesis using a versatile collagen specific probe*. Journal of Structural Biology.

[23] Mees G. (2012). *99mTc-labeled tricarbonyl his-CNA35 as an imaging agent for the detection of tumor vasculature*. Journal of Nuclear Medicine.

[24] Danila D., Johnson E., Kee P. (2013). *CT imaging of myocardial scars with collagen-targeting gold nanoparticles*. Nanomedicine.

[25] Kee P.H., Danila D. (2018). *CT imaging of myocardial scar burden with CNA35-conjugated gold nanoparticles*. Nanomedicine.

[26] Baues M. (2020). *A collagen-binding protein enables molecular imaging of kidney fibrosis in vivo*. Kidney International.

[27] Li F. (2024). *Collagen-Targeting Self-Assembled Nanoprobes for Multimodal Molecular Imaging and Quantification of Myocardial Fibrosis in a Rat Model of Myocardial Infarction*. ACS Nano.

[28] Klink A. (2011). *In vivo characterization of a new abdominal aortic aneurysm mouse model with conventional and molecular magnetic resonance imaging*. Journal of the American College of Cardiology.

[29] Zhou Q. (2019). *Construction of CNA35 Collagen-Targeted Phase-Changeable Nanoagents for Low-Intensity Focused Ultrasound-Triggered Ultrasound Molecular Imaging of Myocardial Fibrosis in Rabbits*. ACS Applied Materials & Interfaces.

[30] Reulen S.W. (2009). *Collagen targeting using protein-functionalized micelles: the strength of multiple weak interactions*. Journal of the American Chemical Society.

[31] Breurken M. (2011). *Collagen targeting using multivalent protein-functionalized dendrimers*. Bioorganic & Medicinal Chemistry.

[32] Helms B.A. (2009). *High-affinity peptide-based collagen targeting using synthetic phage mimics: from phage display to dendrimer display*. Journal of the American Chemical Society.

[33] Hernandez-Rocamora V.M. (2011). *Choline dendrimers as generic scaffolds for the non-covalent synthesis of multivalent protein assemblies*. Chemical Communications (London).

[34] Li Y. (2013). *Direct detection of collagenous proteins by fluorescently labeled collagen mimetic peptides*. Bioconjugate Chemistry.

[35] Flajnik M.F. (2023). *An Ancient MHC-Linked Gene Encodes a Nonrearranging Shark Antibody, UrIg, Convergent with IgG*. Journal of Immunology.

[36] Boudko S.P. (2009). *Crystal structure of human collagen XVIII trimerization domain: A novel collagen trimerization Fold*. Journal of Molecular Biology.

[37] Aper S.J. (2014). *Colorful protein-based fluorescent probes for collagen imaging*. PLoS One1.

[38] Boudko S.P. (2018). *Basement membrane collagen IV: Isolation of functional domains*. Methods in Cell Biology.

[39] Kuhn K. (1981). *Macromolecular structure of basement membrane collagens*. FEBS Letters.

[40] Timpl R. (1981). *A network model for the organization of type IV collagen molecules in basement membranes*. European Journal of Biochemistry.

[41] Oberbaumer I. (1982). *Shape and assembly of type IV procollagen obtained from cell culture*. The EMBO Journal.

[42] Anazco C. (2016). *Lysyl Oxidase-like-2 Cross-links Collagen IV of Glomerular Basement Membrane*. The Journal of Biological Chemistry.

[43] Boudko S.P. (2010). *The NC2 domain of collagen IX provides chain selection and heterotrimerization*. The Journal of Biological Chemistry.

[44] Pedchenko V. (2019). *A chloride ring is an ancient evolutionary innovation mediating the assembly of the collagen IV scaffold of basement membranes*. The Journal of Biological Chemistry.

[45] Itzhaki R.F., Gill D.M. (1964). *A Micro-Biuret Method for Estimating Proteins*. Analytical Biochemistry.

[46] Pettersen E.F. (2021). *UCSF ChimeraX: Structure visualization for researchers, educators, and developers*. Protein Science.

[47] Meng E.C. (2023). *UCSF ChimeraX: Tools for structure building and analysis*. Protein Science.

[48] Goddard T.D. (2018). *UCSF ChimeraX: Meeting modern challenges in visualization and analysis*. Protein Science.

