# Supplementary material for: The staphylococcal collagen adhesin CNA35 effectively detects collagen and its fragments in blots after SDS-PAGE
Source: Matrix Biol Plus. 2025 May 15;26:100174. doi: 10.1016/j.mbplus.2025.100174 (PMC12156248; doi:10.1016/j.mbplus.2025.100174)
Supplement: Supplementary Data 1 [file mmc1.docx]

A

His_6_-Trx-thr-CNA35 (monomer):

MG**HHHHHH**GSGMSDKIIHLTDDSFDTDVLKADGAILVDFWAEWCGPCKMIAPILDEIADEYQGKLTVAKLNIDQNPGTAPKYGIRGIPTLLLFKNGEVAATKVGALSKGQLKEFLDANLAGSGSGLVPRGSGPGSTSGSARDISSTNVTDLTVSPSKIEDGGKTTVKMTFDDKNGKIQNGDTIKVAWPTSGTVKIEGYSKTVSLTVKGEQVGQAVITPDGATITFNDKVEKLSDVSGFAEFEVQGRNLTQTNTSDDKVATITSGNKSTNVTVHKSEAGTSSVFYYKTGDMLPEDTTHVRWFLNINNEKRYVSKDITIKDQIQGGQQLDLSTLNINVTGTHSNYYSGPNAITDFEKAFPGSKITVDNTKNTIDVTIPQGYGSLNSFSINYKTKITNEQQKEFVNNSQAWYQEHGKEEVNGKAFNHTVHNINANAGIEGTVK

His_6_-Trx-thr-18TD-CNA35 (trimer):

MG**HHHHHH**GSGMSDKIIHLTDDSFDTDVLKADGAILVDFWAEWCGPCKMIAPILDEIADEYQGKLTVAKLNIDQNPGTAPKYGIRGIPTLLLFKNGEVAATKVGALSKGQLKEFLDANLAGSGSGLVPRGSGSSGVRLWATRQAMLGQVHEVPEGWLIFVAEQEELYVRVQNGFRKVQLEARTPLPGSGSGTSGSARDISSTNVTDLTVSPSKIEDGGKTTVKMTFDDKNGKIQNGDTIKVAWPTSGTVKIEGYSKTVSLTVKGEQVGQAVITPDGATITFNDKVEKLSDVSGFAEFEVQGRNLTQTNTSDDKVATITSGNKSTNVTVHKSEAGTSSVFYYKTGDMLPEDTTHVRWFLNINNEKRYVSKDITIKDQIQGGQQLDLSTLNINVTGTHSNYYSGPNAITDFEKAFPGSKITVDNTKNTIDVTIPQGYGSLNSFSINYKTKITNEQQKEFVNNSQAWYQEHGKEEVNGKAFNHTVHNINANAGIEGTVK

B


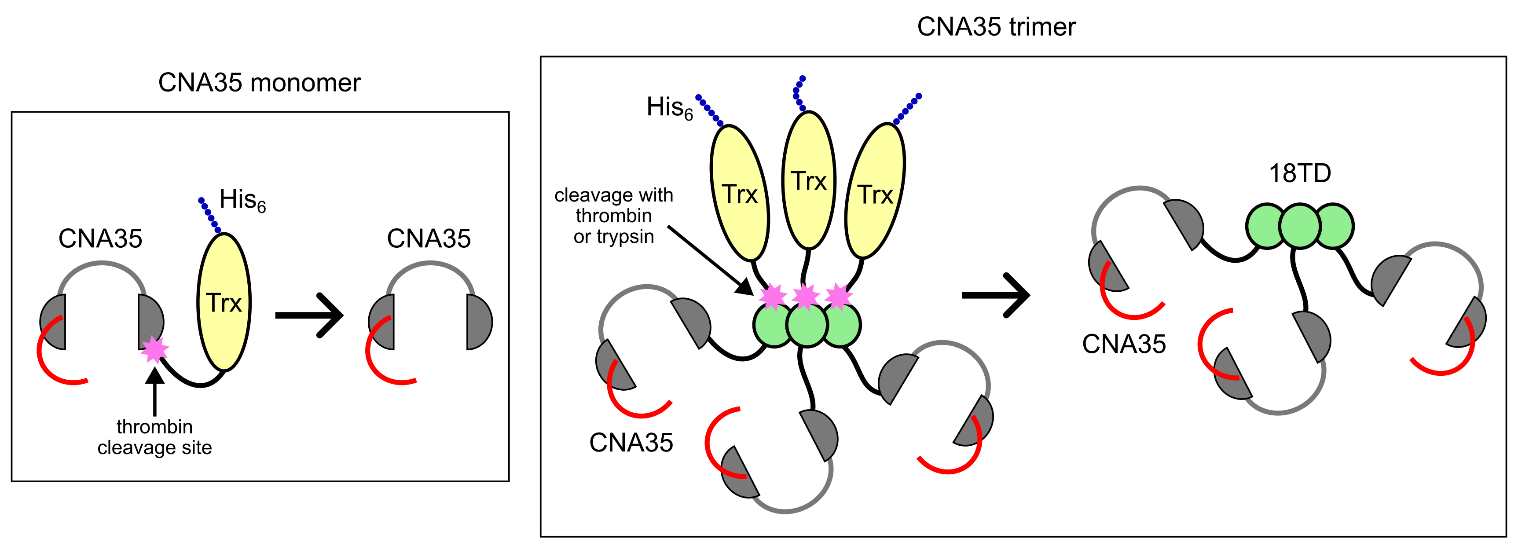


Fig. S1. **Sequences and design of CNA35 fusion constructs.** (A) Protein sequences of the CNA35 monomer and trimer fusion constructs. Trx – *E. coli* thioredoxin sequence, thr – thrombin cleavage sequence, 18TD – sequence of the human collagen XVIII trimerization domain. (B) Schematic design of the CNA35 fusion constructs and their final products.


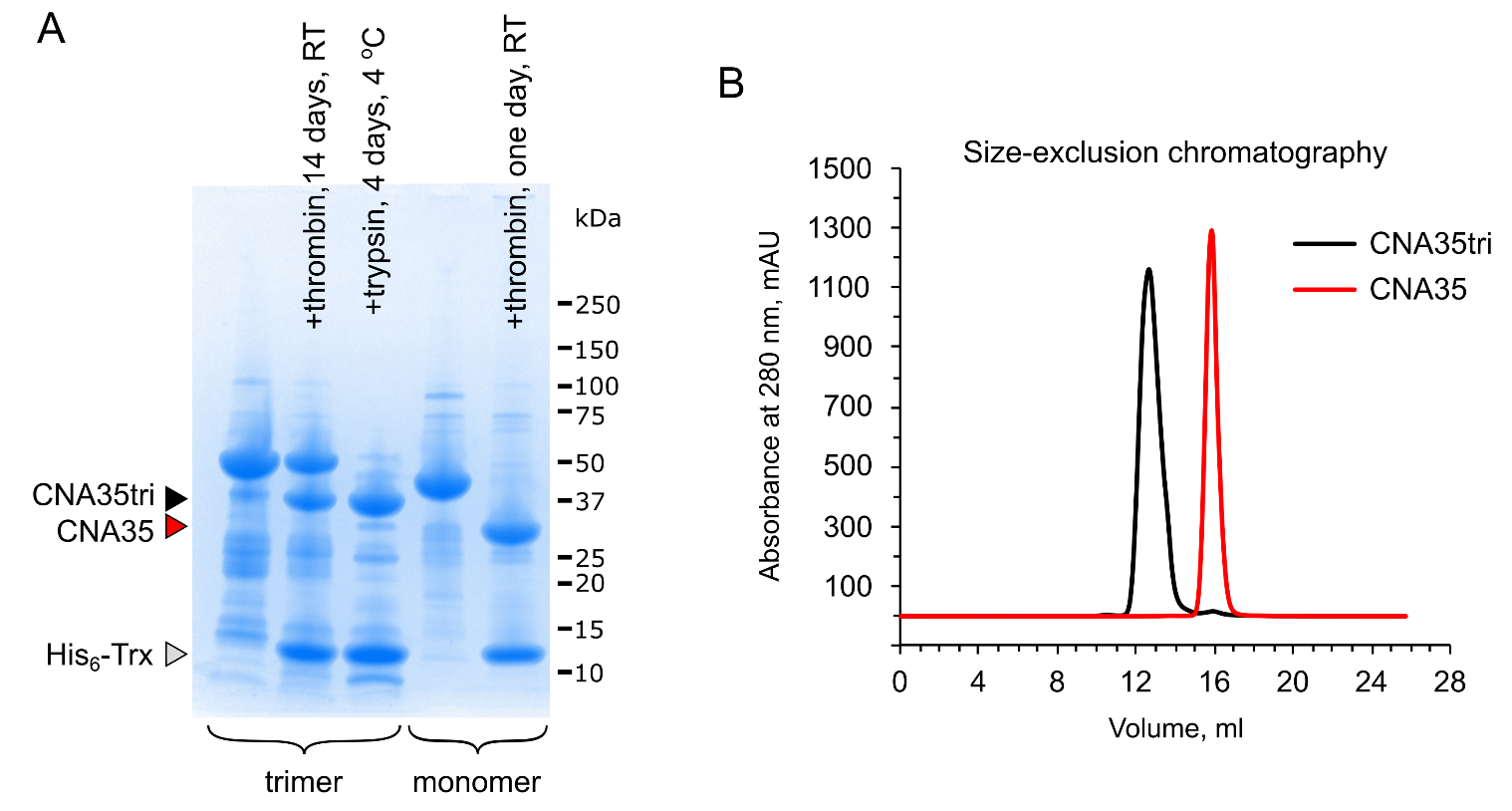


Fig. S2. **Proteolytic cleavage of the fusion constructs and final purification step of the CNA35 trimer and monomer.** (A) The trimeric fusion construct showed only partial cleavage after 14 days at room temperature when treated with thrombin, whereas the monomeric fusion construct was fully cleaved within just one day. In contrast, complete cleavage of the trimeric fusion construct using trypsin was achieved within four days at 4 ºC. Protein samples were analyzed using 4-20% SDS-PAGE and coomassie staining. (B) The final purification of the CNA35 trimer (CNA35tri) and monomer (CNA35) was performed using size-exclusion chromatography.


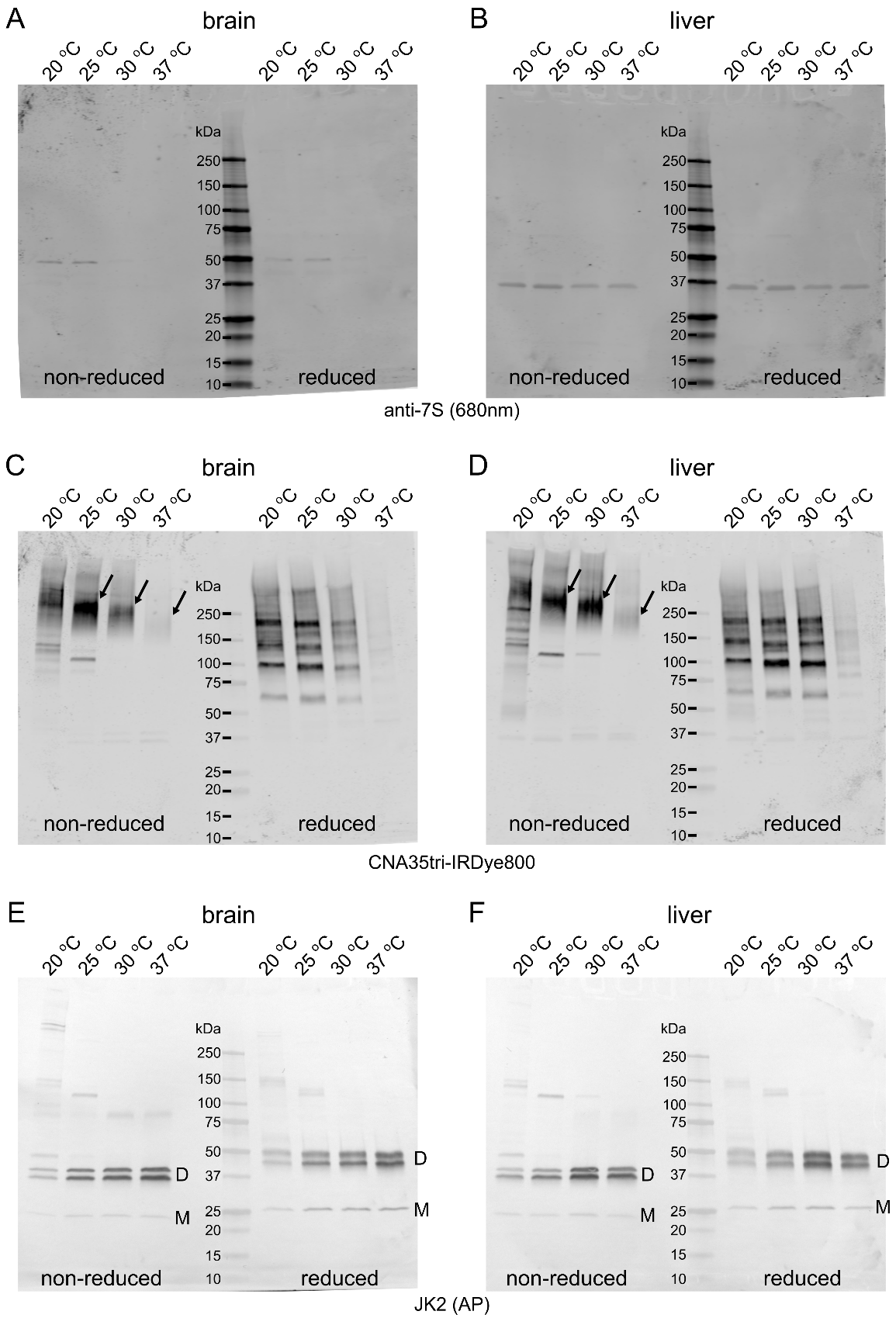


Fig. S3. **Collagenase digest of brain and liver matrix.** Insoluble homogenate of murine brain and liver was subjected to collagenase digest in a suspension at various temperatures and soluble fractions were analyzed on western blots to detect 7S domain (A and B), collagen triple helix (C and D), and the NC1 domain (E and F). A, C, and E show results for brain material. B, D, and F show results for liver material. Amount of collagenase added was 12 units/ml. Amount of material loaded per lane corresponded to 2 mg of initial insoluble homogenate. Arrows indicate 7S domain. Under reducing conditions, the 7S domain breaks down into a series of polypeptides that are crosslinked by non-reducible lysyl-derived bonds. NC1 monomer (designated as “M”) and dimer (designated as “D”) bands are labeled. Anti-7S antibody was C0157 (AssayBioTech). Anti-NC1 antibody was JK2 (from Dr. Y. Sado). 4-15% SDS-PAGE gels were utilized to separate protein bands, and an advanced staining protocol was employed.


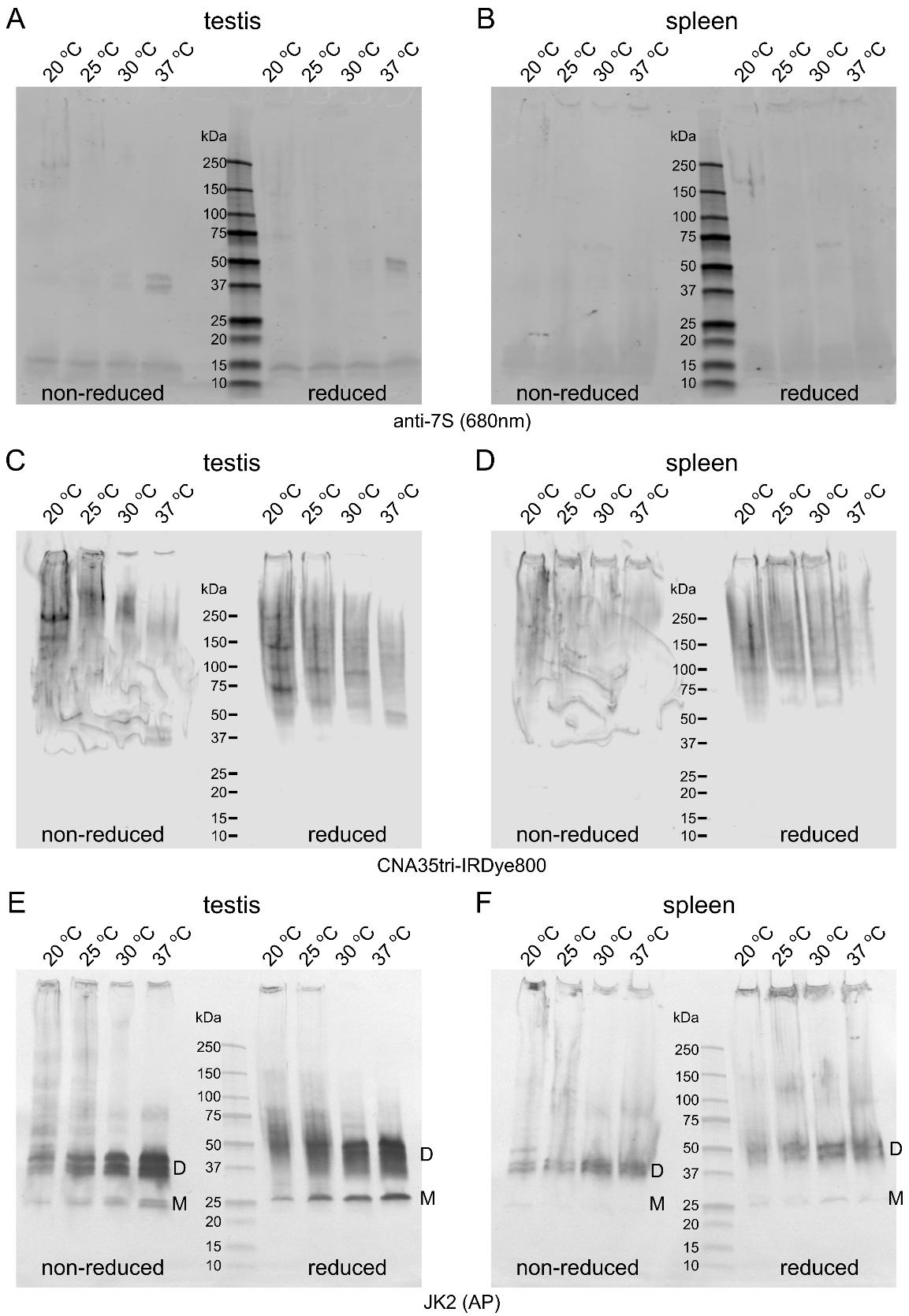


Fig. S4. **Collagenase digest of testis and spleen matrix.** Insoluble homogenate of murine testis and spleen was subjected to collagenase digest in a suspension at various temperatures and soluble fractions were analyzed on western blots to detect 7S domain (A and B), collagen triple helix (C and D), and the NC1 domain (E and F). A, C, and E show results for testis material. B, D, and F show results for spleen material. Amount of collagenase added was 12 units/ml. Amount of material loaded per lane corresponded to 2 mg of initial insoluble homogenate. Arrows indicate 7S domain. NC1 monomer (designated as “M”) and dimer (designated as “D”) bands are labeled. Anti-7S antibody was C0157 (AssayBioTech). Anti-NC1 antibody was JK2 (from Dr. Y. Sado). 4-15% SDS-PAGE gels were utilized to separate protein bands, and an advanced staining protocol was employed.
